# *Kaempferia parviflora* Extract (SIRTMAX^®^) reduces body fat in adults who are overweight: a 12-week randomized, double-blind, placebo-controlled trial

**DOI:** 10.3389/fnut.2025.1576024

**Published:** 2025-06-25

**Authors:** Hyun Kyung Sung, Jinhak Kim, Dongchan Oh, Sang Won Eun, Jin Tatsuzaki, Seon Mi Shin, Su Hyeon Jeong

**Affiliations:** ^1^Department of Education, College of Korean Medicine, Dongguk University, Gyeongju-si, Gyeongsangbuk-do, Republic of Korea; ^2^R&D Division, Daehan Chemtech Co., Ltd., Gwacheon-si, Gyeonggi-do, Republic of Korea; ^3^Tokiwa Phytochemical Co. Ltd., Sakura, Chiba, Japan; ^4^Department of Internal Medicine, College of Korean Medicine, Semyung University, Jecheon, Republic of Korea; ^5^Department of Rehabilitation Medicine of Korean Medicine, Semyung University Chungju Korean Medicine Hospital, Chungju-si, Chungcheongbuk-do, Republic of Korea

**Keywords:** *Kaempferia parviflora*, body weight, obesity, overweight, body fat, fat reduction, randomized controlled trial

## Abstract

**Background/objectives:**

*Kaempferia parviflora* Extract (SIRTMAX^®^) has been reported to alleviate obesity. However, few studies have investigated this topic and none have evaluated its potential among Koreans. Therefore, we aimed to evaluate the efficacy of *K. parviflora* extract in body fat reduction among Korean adults.

**Methods:**

In total, 108 individuals were screened and randomly assigned to the intervention and control groups, and the results of 83 participants were analyzed. Changes from baseline (when the consumption of test and control foods commenced) to the end of the 12th week, were compared. The primary endpoints were body fat mass and body fat percentage changes, measured using dual-energy X-ray absorptiometry. The secondary endpoints were changes in (1) lean body mass; (2) total fat area, subcutaneous fat area, visceral fat area, and visceral subcutaneous fat ratio; (3) body weight, body mass index, waist circumference, hip circumference, and waist-to-hip circumference ratio; (4) serum total cholesterol, low-density lipoprotein cholesterol, triglyceride, high-density lipoprotein cholesterol, leptin, and adiponectin; (5) and body fat mass, body fat percentage, and lean body mass by region (arms, legs, trunk, android, and gynoid).

**Results:**

After 12 weeks, body fat, body weight, and body mass index significantly reduced in the intervention group compared with the control group. Similar findings were observed for total fat area, subcutaneous fat area, visceral fat area, and visceral subcutaneous fat ratio. However, no significant difference was observed in waist and hip circumference values. Additionally, the intervention food was determined to be safe.

**Conclusion:**

This study suggests that health functional foods are effective in reducing body fat, thereby preventing various obesity-related diseases and metabolic syndromes.

## 1 Introduction

Obesity is a major public health issue with a significant impact on society and the medical system, and an increasing prevalence worldwide. Obesity has traditionally been viewed as a problem regarding the lifestyle and eating habits of an individual; however, in the 20th century, it gradually became recognized as a pathological condition, and as obesity rate increased along with changes in eating habits, it became a condition considered to require medical management (1).

The World Health Organization declared obesity an epidemic in 1997 (2). There has been much controversy over whether obesity should be considered a disease (3–7), and until the early 2000s, there was a view that obesity played a role in the progression toward disease (8). However, according to the Nagoya Declaration of 2015, obesity is currently defined as a pathological condition requiring clinical intervention (9). Obesity is defined as an excessive accumulation of adipose tissue in the human body; individuals with body mass index (BMI) of 30 kg/m^2^ and 25–29.9 kg/m^2^ are classified as obese and overweight, respectively. Obesity is a complex, chronic, and recurrent disease caused by the interaction of food, low physical activity, and other environmental factors with genetic susceptibility (9, 10). If not properly managed, it is a risk factor for various diseases such as hyperlipidemia, type 2 diabetes, hypertension, gout, osteoporosis, fatty liver, and obstructive sleep apnea, and it can increase the incidence of serious diseases such as various cancers and premature death, making it a major disease that requires management (11–14). The treatment and management of obesity range from preventive approaches, such as healthy lifestyle habits, diet management, and exercise, to various pharmacological treatments and dietary supplement intake. The US Food and Drug Administration has approved various treatments, such as drugs, surgery, and endoscopic treatment, that can promote 5–35% weight loss (15–17). Dietary supplements are considered an alternative to traditional treatments, including drugs and surgical treatments, owing to their accessibility to the general public and low toxicity. Various products have been developed and utilized, with positive effects through various mechanisms, such as a decrease in fat production, appetite, and nutrient absorption, or increase in fat decomposition and energy consumption (18). *Kaempferia parviflora* (KP), also known as “black ginger,” is a perennial herbaceous plant belonging to the ginger family (Zingiberaceae). It is native to tropical regions of Southeast Asia and South Asia, and in Thai, it is called “Kracahidam,” meaning black finger root; it has been used as a traditional treatment to lower blood sugar, improve blood circulation, improve inflammation, allergies, and gastrointestinal disorders, and increase vitality (19, 20). *In vitro* and *in vivo* experiments on KP extract (SIRTMAX®) have shown that it improves blood flow; has antioxidant, anti-inflammatory, and anti-allergic properties; improves gastric ulcers (21–24); and suppresses weight gain, body fat accumulation, and glucose intolerance (25–27). Trial results have shown that it increases energy consumption and fat utilization (28, 29). There have been experimental studies on the ability of KP extract (SIRTMAX®) to improve obesity-related conditions, and randomized controlled trials (RCT) have been conducted in Japan to investigate the body fat reduction effect in individuals that were overweight and obese. However, only a few studies exist, and to date, none have been conducted among Koreans.

Therefore, the objective of this study was to evaluate the efficacy and safety of KP extract (SIRTMAX®) in reducing body fat mass and visceral adiposity in overweight and mildly obese Korean adults over a 12-week period. This randomized, double-blind, placebo-controlled clinical trial was specifically designed to determine whether KP extract (SIRTMAX®) can produce measurable improvements in total and regional fat distribution using dual-energy X-ray absorptiometry (DEXA) and CT imaging, and to assess its impact on related metabolic biomarkers, while accounting for baseline characteristics such as age and family history of obesity.

## 2 Materials and methods

### 2.1 Study design

**Figure 1:**
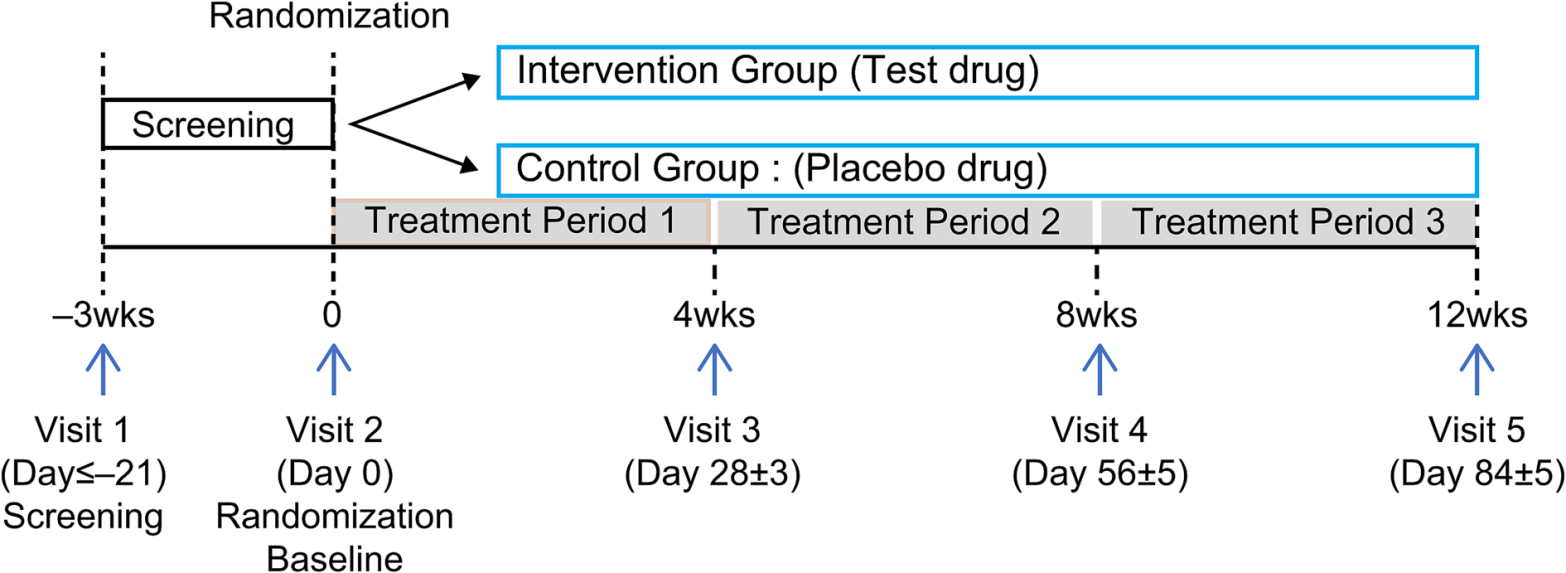
Clinical trial timeline.

This randomized, double-blind, parallel-design, placebo-controlled clinical trial was conducted at Chungju Korean Medicine Hospital of Semyung University (Chungju, Chungcheongbuk-do, Republic of Korea) between February and August 2022. Participants were recruited through the hospital bulletin board and website announcements and were selected according to inclusion and exclusion criteria. One hundred individuals were assigned to either an experimental (*n* = 50) or control (*n* = 50) group, and results were analyzed after 12 weeks of consuming KP extract (SIRTMAX®). The first screening was conducted on February 14, 2022, and the second (baseline visit) on February 23, 2022. Each participant was assigned a screening number in the order of consent provided; they were subsequently screened and diagnosed to determine whether they met the inclusion and exclusion criteria. Those who met the inclusion criteria (*n* = 100) were randomly assigned to the intervention food (KP, *n* = 50) or control food (*n* = 50) group on the second visit. Double-blinding was applied so that neither the researcher nor the participant knew which group they belonged to until the end of the trial. Third, fourth, and fifth screenings were performed 4, 8, and 12 weeks after the baseline screening (Figure 1), respectively. On the baseline, 4-week, and 8-week visit dates, a 33-day supply of the intervention food for human consumption was distributed; the remaining food was retrieved on the 4-week (Visit 3), 8-week (Visit 4), and 12-week (Visit 5) visit dates. The participants’ conditions were assessed at each visit, and a window period of 5 days before and after each visit date was allowed. Laboratory tests (blood and urine tests) and pregnancy tests were performed at Visit 1, and laboratory tests were performed again at Visit 5.

#### 2.1.1 Sample size calculation

The number of participants required for the trial was calculated based on previous studies (30, 31), using the expected difference in the rate of change in body fat mass between the intervention and control groups. The hypotheses for the primary outcome (change in body fat mass) were defined as follows:

- **Null hypothesis (H₀):** There is no difference in the mean change in body fat mass between the intervention group and the control group. That is,

H0:D1t−D1c=0

- **Alternative hypothesis (H₁):** There is a significant difference in the mean change in body fat mass between the intervention group and the control group. That is,

H1:D1t−D1c≠0

Where D₁t represents the change in body fat mass after consuming the intervention food, and D₁c represents the change in body fat mass after consuming the control food.

The results of a previous study involving the use of herbal extract powder were used to calculate the number of participants. According to the results of the study (32), the change in body fat mass in the herbal extract powder group was −1.6 kg, and that in the control food group was −0.1 kg (*p* = 0.023). Using these results, the effect size of the present trial was assumed to be −1.5, with a pooled standard deviation of 2.2676. The formula for calculating the number of participants of the trial for hypothesis testing was as follows:


$$n_{c}=\frac{2{\left(Z_{1-\alpha /2}+Z_{1-\beta }\right)}^{2}\sigma_{1}^{2}}{{\left(D_{1t}-D_{1c}\right)}^{2}}=\frac{2{\left(1.9600+0.9945\right)}^{2}\;2.2676^{2}}{{\left(-1.5\right)}^{2}}\approx 40$$

#### 2.1.2 Statistical method

The results of this study were evaluated based on the per protocol set (PPS). The significance level was set at 5%, and a two-sided test was used. Continuous variables were presented by the number of participants, mean, and standard deviation; categorical variables were presented as frequencies and percentages. For comparison between the intervention and control groups regarding the functional evaluation variables, a general linear model (GLM) was applied, with the change in the outcome value as the response variable and the baseline outcome value, sex, and intake group as independent variables. In cases where the parametric method was not appropriate owing to outliers, the Wilcoxon rank-sum test (a nonparametric method) was applied. Changes before and after intake between the test and control groups were analyzed using the paired t-test or Wilcoxon signed-rank test. The number of participants who developed adverse reactions, adverse drug reactions, and serious adverse reactions; incidence rate; 95% two-sided confidence interval; and number of cases were presented by group. Between-group differences were analyzed using the Chi-squared test or Fisher’s exact test.

#### 2.1.3 Selection criteria

1. Age range: 19–60 years.
2. BMI of 25–30 kg/m^2^.
3. Voluntary participation with signed written consent form.

#### 2.1.4 Exclusion criteria

1. Severe cerebrovascular disease (cerebral infarction, cerebral hemorrhage, etc.), heart disease (angina pectoris, myocardial infarction, heart failure, and arrhythmia requiring treatment), or malignant tumors within the prior 6 months (however, those with a history of cerebrovascular disease or heart disease but who were considered clinically stable were able to participate in the study at the investigator’s discretion).
2. Use of medications that affect body weight (fat absorption inhibitors, appetite suppressants, health foods/supplements related to improving obesity, and psychiatric drugs for depression, beta-blockers, diuretics, contraceptives, steroids, and female hormones) within the prior 1 month.
3. Obesity or overweight due to endocrine diseases such as hypothyroidism or Cushing’s syndrome.
4. Undergoing continuous treatment or using medication for gastrointestinal disorders (gastric ulcers, chronic digestive disorders, irritable bowel syndrome, etc.)
5. Significant psychiatric history or current illness (schizophrenia, epilepsy, anorexia, hyperphagia, etc.) or a history of alcohol or other drug abuse.
6. Inability to exercise due to musculoskeletal disorders.
7. Fasting blood sugar levels ≥126 mg/dL, random blood sugar levels of 200 mg/dL, or use of oral hypoglycemic agents or insulin for diabetes treatment.
8. Uncontrolled hypertension (blood pressure ≥160/100 mmHg, measured after a 10-min rest).
9. Use of medication for dyslipidemia.
10. Aspartate aminotransferase (glutamic oxaloacetic transaminase) or alanine aminotransferase (glutamic pyruvic transaminase) levels ≥2.5 times the upper limit of normal in the testing institution.
11. Creatinine levels ≥2 times the upper limit of normal in the testing institution.
12. Loss of >5% of body weight within the prior 3 months.
13. Participation in a commercial obesity program within the prior 3 months.
14. Participation in similar trials within the prior 6 months.
15. Pregnancy or breastfeeding during the trial.
16. Allergic reactions to foods used in the trial.
17. Being unsuitable as determined by the researcher for other reasons.

### 2.2 Ethical considerations

This RCT was approved by the Institutional Review Board (IRB) of Semyung University Chungju Korean Medicine Hospital and conducted in compliance with the IRB’s approval and relevant regulations, such as the Helsinki Declaration and Korean Good Clinical Practice (KGCP). The purpose of the trial and characteristics of the intervention food were explained to the participants. Only volunteers who knew the purpose and risks of the RCT and provided a written consent form participated in the trial. Participants were informed that they could withdraw their consent to participate in the RCT at any time during the study, and that compensation for victims would be provided. They were also informed that the results obtained during the study period would be anonymized and recorded in an electronic case report form (IRB No. SMCJH 2201-01).

### 2.3 Randomization

Block randomization was used to ensure fair allocation. The research team judged the participants to be suitable for participation in the trial based on the selection and exclusion criteria. They were assigned to either the intervention food or control food group at a 1:1 ratio. The randomization table was sequentially applied from participant number 1 of the trial by permuting random numbers generated by the randomization program of the SAS® system. After labeling the food packaging for the trials according to the randomization table, it was supplied to the institution conducting the trial; the packaging material was printed identically, except for the individual IP number, so that the group could not be identified. Therefore, the participants of the trial, researchers of the trial, intervention food manager, research nurse, and trial monitor were blinded. The randomization code and IP number were managed by a blinding manager who was not affiliated with the trial on the side of the trial sponsor, and were not disclosed until statistical analysis, except in cases where it was absolutely necessary to view the code due to a serious medical emergency. The intervention food manager supplied the trial food with the assigned IP number to the relevant trial participant; in the case of loss or damage of the trial food, the IP number was reassigned and maintained through the interactive web response system.

### 2.4 Intake of intervention and control foods

The intervention food used in the RCT contained 100 mg of the main ingredient [KP extract (SIRTMAX®) (20.0%)], one capsule (500 mg) of excipients, maltodextrin (77.0%), magnesium stearate (1.0%), and silicon dioxide (2.0%). The control food contained one capsule (500 mg) of excipients, maltodextrin (97.0%), magnesium stearate (1.0%), and silicon dioxide (2.0%). The main ingredient in the intervention food, KP extract (SIRTMAX®), a commercial product sold by TOKIWA PHYTOCHEMICAL CO., LTD. in Japan—which contains 50% KP ethanol extract. Therefore, one capsule of this intervention food contained 50 mg of KP ethanol extract. The intake method involved ingesting one capsule daily before breakfast with sufficient water for 12 weeks. Intervention and control foods were manufactured in similar shapes, colors, and sizes. Participants received 33-day doses at Visits 2, 3, and 4, and then returned the remaining doses on the next visits (Visits 3, 4, and 5) to evaluate overall drug compliance. Participants were asked to maintain their usual lifestyle habits (exercise and diet) during the participation period but were prohibited from consuming drugs or health functional foods that could cause body fat reduction. Exercise, diet, and medications (excluding drugs that could cause body fat reduction) that had been maintained prior to participation were permitted at the discretion of the researcher. If any new medications were taken during the treatment period, the reason, dosage, medication duration, and subsequent status were recorded at each visit. Adverse reactions were checked at each visit: participation in the study was discontinued if there were serious adverse reactions to medications or treatments that could affect body fat and lipid levels; if it was difficult to continue the trial because the food for RCT was not consumed according to the dosage and directions; if consent to participate in the study was withdrawn; or if other follow-up measures were difficult (violation of the visit schedule, the participant’s personal schedule, etc.).

### 2.5 Evaluation items

The primary evaluation variables were changes in body fat mass (g) and percentage (%) measured using DEXA from Visit 2 (baseline) to Visit 5 (12 weeks later). The secondary evaluation variables were as follows: (1) change in lean body mass (g) measured using DEXA after 12 weeks compared with the baseline; (2) change in total fat area (cm^2^), subcutaneous fat area (cm^2^), visceral fat area (cm^2^), and visceral subcutaneous fat ratio (VSR) measured using abdominal computed tomography (CT) after 12 weeks compared with the baseline; (3) change in body weight (kg) after 4, 8, and 12 weeks compared with the baseline; (4) change in BMI (kg/m^2^) after 4, 8, and 12 weeks compared with the baseline; (5) change in waist circumference (cm), hip circumference (cm), and waist/hip ratio after 4, 8, and 12 weeks compared with the baseline; (6) blood lipid concentrations (total cholesterol) and low-density lipoprotein (LDL) cholesterol; (7) changes in leptin (ng/mL) and adiponectin (ng/mL) after 12 weeks compared with the baseline; and (8) changes in body fat mass (g), body fat percentage (%), and lean body mass (g) by region (arms, legs, trunk, android, and gynoid) using DEXA after 12 weeks compared with the baseline. DEXA measurements were performed using dedicated equipment (GE Healthcare) and Lunar Prodigy Advance software at Visits 2 and 5, and the amount of fat present in the chest, abdomen, and pelvis, surrounded by a virtual boundary line dividing the head and limbs was calculated. Measurements were performed using abdominal CT scans at Visits 2 and 5. The visceral and total abdominal fat areas were measured with Hounsfield units (HU) ranging from −190 to −30, based on the 4th and 5th lumbar vertebrae on the abdominal CT image. The abdominal and back muscles were divided into visceral fat tissue on the inside and abdominal subcutaneous fat tissue on the outside, and the abdominal subcutaneous fat area was calculated by subtracting the visceral fat area from the total fat area.

### 2.6 Safety evaluation

#### 2.6.1 Investigation of adverse reactions and vital signs

Participants voluntarily reported adverse reactions during the RCT from Visit 2 to Visit 5. In the case of adverse reactions, the date of onset and disappearance, degree and result of the adverse reaction, measures taken in relation to the food for RCT, causal relationship with the food, name of the suspected drug other than the food for RCT, and whether the treatment for the adverse reaction was administered were investigated. Additionally, vital signs (systolic blood pressure, diastolic blood pressure, pulse, and body temperature) were measured at each visit to determine the subject’s condition.

#### 2.6.2 Laboratory tests

Blood and urine tests were performed at Visit 1 (screening) and Visit 5 (final). If 2 weeks had passed since the screening test, or if there were missing items or abnormal results, retests were conducted for those items. Additional tests were conducted if there were adverse reactions or if the investigator determined that testing was necessary. All women of childbearing age, except those who were menopausal or medically incapable of pregnancy, underwent a pregnancy test (urine hCG). Table 1 lists the test items used in this study.

**Table 1:** Laboratory test items.

Division	Items
Hematological tests	Hemoglobin, hematocrit, platelet, WBC, RBC
Blood chemistry test	Total cholesterol, HDL cholesterol, LDL cholesterol, triglyceride, glucose, BUN, creatinine, ALT, AST, γ-GT, total bilirubin, albumin, total protein
Urine test	Specific gravity, pH, protein, glucose, blood (RBC), urine hCG (pregnancy test conducted, if necessary)

HDL, high-density lipoprotein; LDL, low-density lipoprotein; BUN, blood urea nitrogen; ALT, alanine transferase; AST, alanine aspartate; γ-GT, gamma-glutamyl transferase; RBC, red blood cell.

### 2.7 Lifestyle survey

At Visits 2 (baseline) and 5 (final), the participants’ smoking, drinking, eating, and exercise habits were investigated. Smoking status was assessed by examining whether they smoked, smoking history, current smoking amount, and smoking period. Drinking status was assessed by examining whether they drank alcohol and the amount of alcohol they drank over the past week. Eating habits were assessed by examining how regular their meals were using the 24-h recall method, after which the total calories consumed were calculated using the Canpro program (ver. 5.0). Exercise habits were assessed by examining whether they exercised over the past week and the number of times they exercised, using the International Physical Activity Questionnaire.

## 3 Results

### 3.1 Participant characteristics

**Figure 2:**
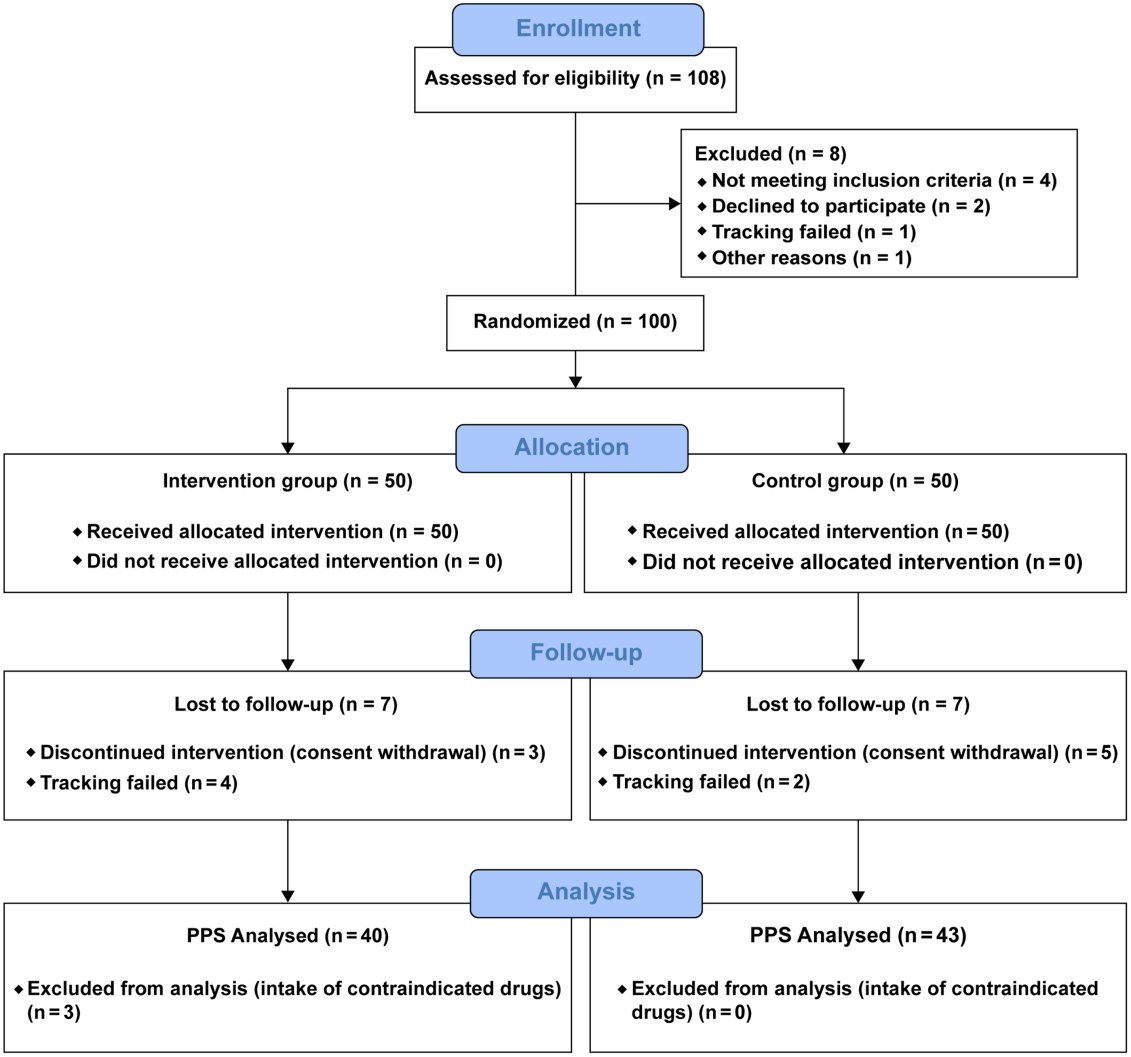
Flow chart of clinical trial.

**Figure 3:**
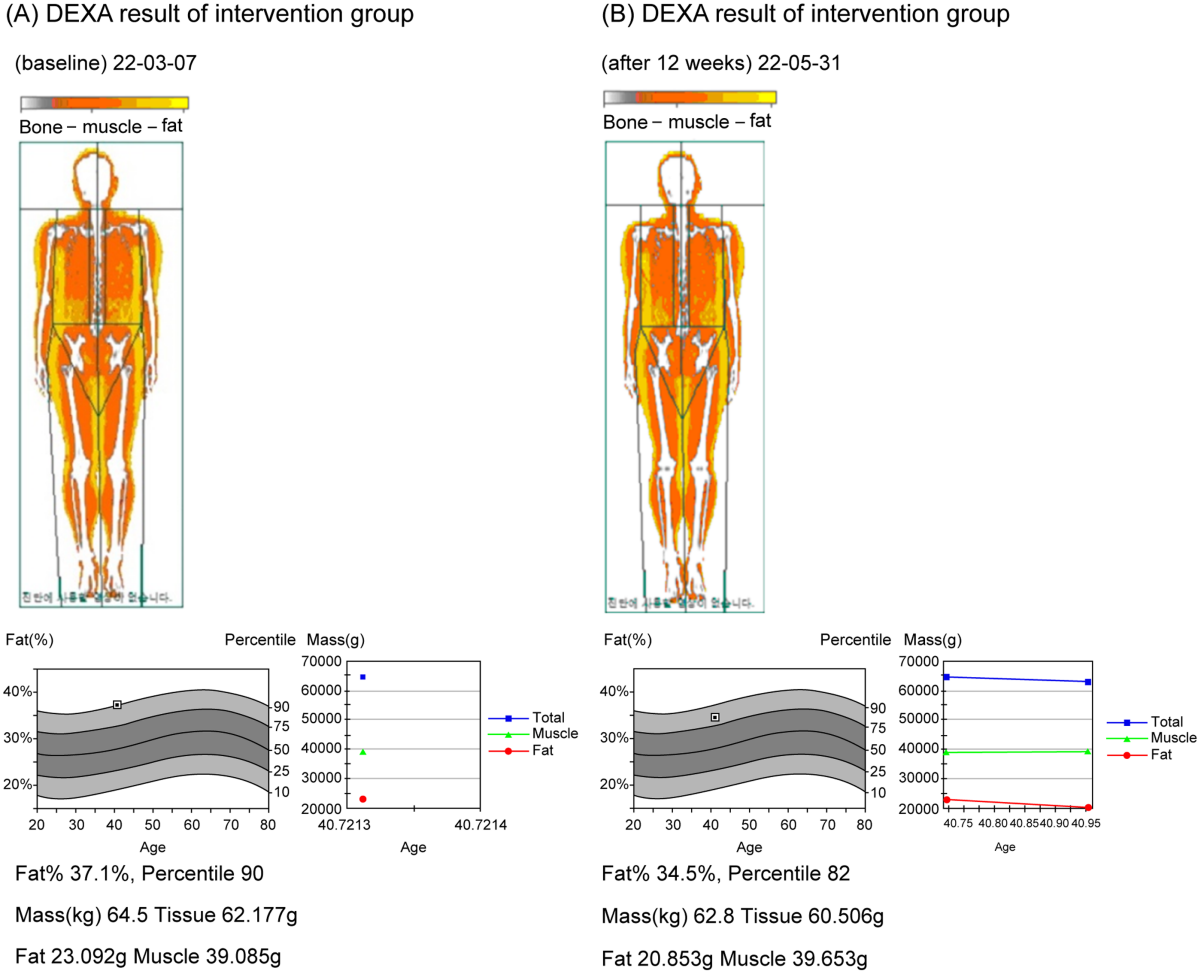
DEXA images before and after participation of the trial group. **(A)** DEXA image before (22-03-07); **(B)** DEXA image after 12 weeks (22-05-31).

**Figure 4:**
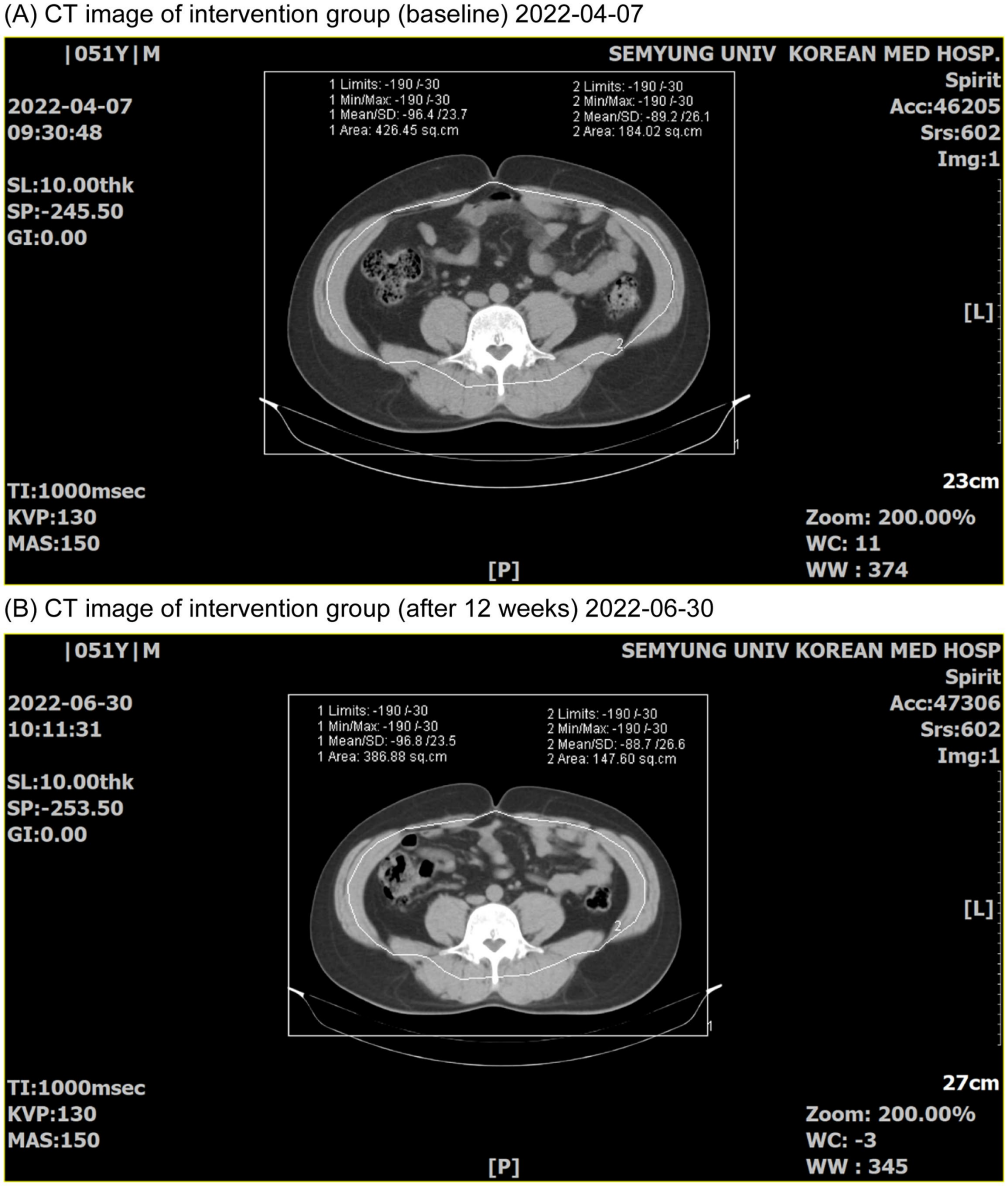
CT images before and after participation in the trial group. **(A)** CT image before (2022-04-07); **(B)** CT image after 12 weeks (2022-06-30).

In total, 108 individuals were screened, of whom eight were excluded and 100 were randomly assigned to the KP intervention group (*n* = 50) and placebo control group (*n* = 50). Fourteen participants dropped out during the trial, and three were excluded from the analysis (Figure 2). Therefore, the results of 83 participants were analyzed. Age, sex, height, weight, BMI, waist/hip circumference, lean body mass, and family history of obesity were compared between the intervention and control groups (Table 2). DEXA and CT findings are shown in Figures 3, 4, respectively. Mean age was significantly higher in the intervention group than in the control group (46.30 ± 9.30 years vs. 43.23 ± 7.92 years; *p* < 0.05). Additionally, the number of people with a family history of obesity was significantly higher in the intervention group than in the control group (22 vs. 14; *p* < 0.05). No statistically significant differences were observed in the other variables.

**Table 2:** Participant characteristics.

Items		Group		
		Intervention group (*n* = 40)	Control group (*n* = 43)	*p*-value
Gender	Male	*n* = 10	*n* = 11	0.9515^1^
	Female	*n* = 30	*n* = 32	
Age		46.30 ± 9.30	43.23 ± 7.92	0.0354^2^
Height (cm)		163.66 ± 8.58	162.76 ± 8.51	0.6421^2^
Weight (kg)		73.46 ± 9.69	72.21 ± 8.56	0.5860^2^
Body mass index (kg/m^2^)		27.32 ± 1.50	27.19 ± 1.58	0.7095^2^
Body fat mass (g)		26,408.63 ± 4,182.89	25,367.09 ± 3,567.12	0.2248^2^
Lean body mass (g)		46,857.10 ± 8,681.78	46,633.07 ± 8,233.85	0.8027^2^
Waist circumference (cm)		92.71 ± 5.59	91.23 ± 5.04	0.2108^2^
Hip circumference (cm)		102.09 ± 4.09	102.34 ± 4.62	0.7918^2^
Family history of obesity	Yes	*n* = 22	*n* = 14	0.0393^1^
	No	*n* = 18	*n* = 29	

1 Compared between groups using the Chi-squared test or Fisher’s exact test.

2 Compared between groups using the Unpaired *t*-test or Wilcoxon rank-sum test.

### 3.2 Results of primary evaluation variables

Changes in body fat mass and body fat percentage measured using DEXA, the primary evaluation variables of the RCT, are shown in Table 3. Body fat mass decreased significantly after the trial compared with before in the intervention group (*p* < 0.05); the opposite was observed in the control group (*p* < 0.05). No significant change was observed in body fat percentage before and after the trial in the intervention group, while the control group exhibited a significant increase after the trial (*p* < 0.05); however, the difference between the intake groups was not statistically significant.

**Table 3:** Changes in body fat mass, body fat percentage, and lean body mass using DEXA (V2–V5).

Items			Groups	Visit	Intervention group (*n* = 40)	Control group (*n* = 43)
Body fat mass (g)	V2	26,408.63 ± 4,182.89	25,367.09 ± 3,567.12
	V5	26,070.98 ± 4,136.08	25,671.81 ± 3,565.91
	V2–V5	−337.65 ± 1,018.09	304.72 ± 889.64
	*p*-value	0.0425^1^	0.0300^1^
		0.0054^2^	
		0.0065^3^	
Body fat percentage (%)	V2	37.55 ± 5.40	36.73 ± 5.29
	V5	37.55 ± 5.53	37.13 ± 5.13
	V2–V5	0.00 ± 1.13	0.40 ± 0.95
	*p*-value	0.9889^1^	0.0083^1^
		0.1014^2^	
		0.0938^3^	
Lean body mass (g)	V2	46,857.10 ± 8,681.78	46,633.07 ± 8,233.85
	V5	46,268.68 ± 8,521.38	46,336.12 ± 8,009.98
	V2–V5	−588.43 ± 960.44	−296.95 ± 901.09
	*p*-value	0.0004^1^	0.0364^1^
		0.1599^2^	

1 Compared within groups using the Unpaired *t*-test.

2 Compared between groups using the GLM, adjusted for baseline value and sex.

3 Compared between groups using the GLM, adjusted for baseline value, sex, family history of obesity, and age.

Age and family history of obesity differed between the two intake groups; therefore, they were added as covariates, and changes in body fat mass and body fat percentage before (0 weeks) and after (12 weeks) intake were evaluated. Regarding change in body fat mass, the GLM—including baseline value (body fat mass at Visit 2) and sex as covariates—demonstrated that body fat mass significantly decreased in the intervention food group compared with the control group (*p* = 0.0054). A similar result was observed after adding age and family history of obesity as covariates (*p* = 0.0065). Regarding change in body fat percentage, the GLM—including baseline value (body fat percentage at Visit 2) and sex as covariates—did not demonstrate a statistically significant difference between the intervention and control food groups (*p* = 0.1014). A similar finding was observed after adding age and family history of diabetes as covariates (*p* = 0.0938); therefore, we confirmed that baseline differences in these factors did not affect the functionality evaluation.

### 3.3 Results of secondary evaluation variables

Lean body mass significantly reduced after the trial in both the intervention and control groups (*p* < 0.05); however, no statistically significant difference was observed between the intake groupsTable 2). Total fat area, subcutaneous fat area, visceral fat area, and VSR significantly decreased after the trial in the intervention group (*p* < 0.05), while no significant change was observed in the control group. Between-group comparisons demonstrated a more significant decrease in the intervention group than in the control group (Table 4). Body weight significantly decreased after the trial in the intervention group (*p* < 0.05), while no significant difference was observed in the control group. Between-group comparisons revealed a more significant decrease in the intervention group than in the control group (*p* < 0.05). BMI significantly decreased after the trial in the intervention group (*p* < 0.05), while no significant difference was observed in the control group. Between-group comparisons revealed a more significant decrease in the intervention group than in the control group (*p* < 0.05). Waist circumference decreased significantly after the trial in both groups (*p* < 0.05). Hip circumference decreased significantly after the trial in both groups, with no significant between-group difference. The waist-to-hip circumference ratio showed no significant change after the trial in both groups, with no significant between-group difference (Table 5).

**Table 4:** Changes in total fat area, subcutaneous, visceral fat area, and VSR using CT (V2–V5).

Items			Groups	Visit	Intervention group (*n* = 40)	Control group (*n* = 43)
Total fat area (cm^2^)	V2	386.51 ± 73.23	357.41 ± 62.54
	V5	367.44 ± 67.04	362.01 ± 65.47
	V2–V5	−19.07 ± 30.25	4.59 ± 28.96
	*p*-value	0.0003^1^	0.3042^1^
		0.0025^2^	
Subcutaneous fat area (cm^2^)	V2	270.01 ± 64.29	252.48 ± 60.01
	V5	262.10 ± 59.91	256.66 ± 63.70
	V2–V5	−7.91 ± 23.08	4.18 ± 22.09
	*p*-value	0.0364^1^	0.2212^1^
		0.0332^2^	
Visceral fat area (cm^2^)	V2	116.50 ± 40.59	104.93 ± 34.50
	V5	105.34 ± 34.22	105.34 ± 31.40
	V2–V5	−11.16 ± 16.24	0.41 ± 13.36
	*p*-value	<0.0001^1^	0.8412^1^
		0.0005^2^	
Visceral subcutaneous fat ratio	V2	0.46 ± 0.22	0.45 ± 0.25
	V5	0.43 ± 0.20	0.45 ± 0.22
	V2–V5	−0.04 ± 0.07	−0.01 ± 0.08
	*p*-value	0.0062^1^	0.9234^1^
		0.0242^2^	

1 Compared within groups using the Unpaired *t*-test or Wilcoxon signed-rank test.

2 Compared between groups using the GLM adjusted for baseline and sex or Wilcoxon rank sum test.

**Table 5:** Change in weight, BMI, waist circumference, hip circumference, and waist to hip ratio (V2 to V5).

Items			Groups	Visit	Intervention group (*n* = 40)	Control group (*n* = 43)
Weight (kg)	V2	73.46 ± 9.69	72.21 ± 8.56
	V5	72.38 ± 9.36	72.09 ± 8.54
	V2–V5	−1.08 ± 1.26	−0.12 ± 1.36
	*p*-value	<0.0001^1^	0.5791^1^
		0.0021^2^	
BMI (kg/m^2^)	V2	27.32 ± 1.50	27.19 ± 1.58
	V5	26.92 ± 1.48	27.16 ± 1.73
	V2–V5	−0.39 ± 0.45	−0.03 ± 0.52
	*p*-value	<0.0001^1^	0.7249^1^
		0.0011^2^	
Waist circumference (cm)	V2	92.71 ± 5.59	91.23 ± 5.04
	V5	91.35 ± 5.72	90.55 ± 4.66
	V2–V5	−1.36 ± 2.54	−0.69 ± 2.81
	*p*-value	0.0017^1^	0.0410^2^
		0.3197^2^	
Hip circumference (cm)	V2	102.09 ± 4.09	102.34 ± 4.62
	V5	100.95 ± 3.90	101.47 ± 4.38
	V2–V5	−1.14 ± 2.05	−0.87 ± 1.81
	*p*-value	0.0011^1^	0.0029^1^
		0.4610^2^	
Waist to hip ratio	V2	0.91 ± 0.05	0.89 ± 0.03
	V5	0.90 ± 0.05	0.89 ± 0.03
	V2–V5	−0.00 ± 0.03	0.00 ± 0.02
	*p*-value	0.4163^1^	0.4958^2^
		0.3831^1^	

1 Compared within groups using the Unpaired *t*-test or Wilcoxon signed-rank test.

2 Compared between groups using the GLM adjusted for baseline and sex or Wilcoxon rank sum test.

Total cholesterol levels decreased slightly in the intervention group and increased slightly in the control group before and after the trial; however, these changes were not statistically significant, nor was the difference between groups (Table 6). LDL cholesterol levels decreased slightly in both the intervention group and control group before and after the trial; however, these differences were not statistically significant in either group, nor was the difference between groups (Table 6). Triglyceride levels decreased slightly in the intervention group and increased slightly in the control group before and after the trial; however, these differences were not statistically significant, nor was the difference between groups (Table 6). High-density lipoprotein(HDL) cholesterol levels decreased slightly in both the intervention group and control group before and after the trial, with no statistically significant difference between periods or groups (Table 6). Leptin levels decreased slightly in the intervention group and increased slightly in the control group before and after the trial, with no statistically significant difference between periods or groups (Table 6). Adiponectin levels decreased slightly in both the intervention and control groups before and after the trial, with no statistically significant difference between periods or groups (Table 6).

**Table 6:** Changes in total cholesterol, LDL cholesterol, triglyceride, HDL cholesterol, leptin, and adiponectin levels (V2 to V5).

Items			Groups	Visit	Intervention group (*n* = 40)	Control group (*n* = 43)
Total cholesterol (mg/dL)	V2	216.78 ± 45.43	204.79 ± 33.16
	V5	210.55 ± 40.39	206.28 ± 40.99
	V2–V5	−6.23 ± 24.61	1.49 ± 33.59
	*p*-value	0.0755^1^	0.6625^1^
		0.4041^2^	
LDL cholesterol (mg/dL)	V2	142.53 ± 41.18	132.23 ± 29.58
	V5	137.08 ± 38.18	131.51 ± 36.44
	V2–V5	−5.45 ± 24.41	−0.72 ± 29.98
	*p*-value	0.1611^1^	0.3709^1^
		0.6550^2^	
Triglyceride (mg/dL)	V2	166.03 ± 215.92	143.79 ± 97.83
	V5	150.45 ± 101.63	167.63 ± 100.24
	V2–V5	−15.58 ± 186.64	23.84 ± 95.41
	*p*-value	0.8225^1^	0.0916^1^
		0.2904^2^	
HDL cholesterol (mg/dL)	V2	52.73 ± 12.81	53.40 ± 11.35
	V5	52.43 ± 12.59	52.00 ± 11.99
	V2–V5	−0.30 ± 6.82	−1.40 ± 7.53
	*p*-value	0.7825^1^	0.2310^1^
		0.5385^2^	
Leptin (ng/mL)	V2	21.02 ± 10.48	18.28 ± 9.48
	V5	20.33 ± 14.53	20.42 ± 11.96
	V2–V5	−0.70 ± 8.60	2.14 ± 6.96
	*p*-value	0.6113^1^	0.1530^1^
		0.0748^2^	
Adiponectin (ng/mL)	V2	9,049.98 ± 4,817.34	9,184.72 ± 3,795.77
	V5	8,558.65 ± 4,381.59	8,948.69 ± 4,147.98
	V2–V5	−491.33 ± 2,278.90	−236.03 ± 1,980.32
	*p*-value	0.3709^1^	0.4388^1^
		0.8589^2^	

1 Compared within groups using the Unpaired *t*-test or Wilcoxon signed-rank test.

2 Compared between groups using the GLM adjusted for baseline and sex or Wilcoxon rank sum test.

Body fat mass decreased in the arm region in both the intervention and control groups after the trial, with no statistically significant difference between periods or groups. In the leg region, body fat mass increased slightly in both the intervention and control groups after the trial, with no statistically significant difference between periods or groups (Table 7). In the trunk region, body fat mass decreased in the intervention group after the trial, with no statistical significance; however, it increased significantly in the control group (*p* < 0.05; Table 7). A significant difference was observed between the intervention and control groups (*p* < 0.05). In the android area, body fat mass decreased significantly in the intervention group after the trial (*p* < 0.05), whereas it increased in the control group; however, this change was not significantly different (Table 7). Between-group comparisons revealed a more significant decrease in the intervention group than in the control group (*p* < 0.05). In the gynoid area, body fat mass decreased significantly in the intervention group after the trial (*p* < 0.05); however, this change was not significantly different (Table 7). Between-group comparisons revealed a more significant decrease in the intervention group than in the control group. Body fat percentage(%) increased somewhat in the arms, legs, trunk, and gynoid areas in both the intervention and control groups after the trial, with no significant difference between periods or groups. In the android area, a slight decrease and increase were observed after the trial in the intervention and control groups, respectively, with no statistical significance in either group. Between-group comparisons revealed no significant difference.

**Table 7:** Changes in body fat mass, body fat percentage, lean body mass by area (V2–V5).

**Items**			Groups	Visit	Intervention group (*n* = 40)	Control group (*n* = 43)
Body fat mass (g), arms	V2	2,652.00 ± 597.27	2,609.21 ± 510.59
	V5	2,596.30 ± 563.43	2,565.53 ± 482.69
	V2–V5	−55.70 ± 226.26	−43.67 ± 189.18
	*p*-value	0.1276^1^	0.4077^1^
		0.6098^2^	
Body fat mass (g), legs	V2	7,229.83 ± 1,974.37	7,525.65 ± 1,922.03
	V5	7,238.45 ± 1,727.42	7,557.81 ± 1,904.30
	V2–V5	8.63 ± 657.44	32.16 ± 374.36
	*p*-value	0.5170^1^	0.5762^1^
		0.4385^2^	
Body fat mass (g), trunk	V2	15,499.38 ± 2,612.94	14,304.37 ± 2,017.39
	V5	15,296.08 ± 2,683.68	14,622.51 ± 2,132.43
	V2–V5	−203.30 ± 771.79	318.14 ± 706.95
	*p*-value	0.1037^1^	0.0052^1^
		0.0035^2^	
Body fat mass (g), android	V2	2,565.08 ± 535.26	2,316.79 ± 398.93
	V5	2,487.43 ± 519.51	2,370.23 ± 445.99
	V2–V5	−77.65 ± 174.31	53.44 ± 174.30
	*p*-value	0.0076^1^	0.0508^1^
		0.0027^2^	
Body fat mass (g), gynoid	V2	4,124.53 ± 945.69	4,166.44 ± 927.36
	V5	4,055.03 ± 892.79	4,242.77 ± 927.24
	V2–V5	−69.50 ± 184.80	76.33 ± 264.86
	*p*-value	0.0224^1^	0.0657^1^
		0.0038^2^	
Body fat percentage (%), arms	V2	35.86 ± 8.00	35.42 ± 7.89
	V5	35.94 ± 8.06	35.67 ± 7.68
	V2–V5	0.07 ± 1.85	0.25 ± 1.28
	*p*-value	0.7665^1^	0.2077^1^
		0.9093^2^	
Body fat percentage (%), legs	V2	33.17 ± 7.31	33.71 ± 7.29
	V5	33.23 ± 7.04	33.83 ± 6.92
	V2–V5	0.05 ± 1.24	0.12 ± 1.23
	*p*-value	0.7896^1^	0.5212^1^
		0.7302^2^	
Body fat percentage (%), trunk	V2	42.10 ± 5.25	40.58 ± 4.64
	V5	42.14 ± 5.46	41.16 ± 4.74
	V2–V5	0.04 ± 1.52	0.59 ± 1.34
	*p*-value	0.8768^1^	0.0063^1^
		0.1186^2^	
Body fat percentage (%), android	V2	44.95 ± 5.82	43.24 ± 5.04
	V5	44.63 ± 6.30	43.78 ± 5.26
	V2–V5	−0.32 ± 2.13	0.53 ± 1.96
	*p*-value	0.3436^1^	0.0805^1^
		0.0771^2^	
Body fat percentage (%), gynoid	V2	37.54 ± 7.70	37.98 ± 7.46
	V5	37.61 ± 7.84	38.65 ± 7.26
	V2–V5	0.07 ± 1.61	0.67 ± 1.91
	*p*-value	0.7924^1^	0.0272^1^
		0.1165^2^	
Lean body mass (g), arms	V2	5,159.35 ± 1,298.91	5,197.53 ± 1,304.93
	V5	5,044.53 ± 1,270.34	5,069.72 ± 1,298.94
	V2–V5	−114.83 ± 306.62	−127.81 ± 235.05
	*p*-value	0.0229^1^	0.0009^1^
		0.8601^2^	
Lean body mass (g), legs	V2	15,728.30 ± 3,376.83	15,775.14 ± 3,324.56
	V5	15,578.55 ± 3,357.18	15,690.21 ± 3,064.14
	V2–V5	−149.75 ± 535.69	−84.93 ± 618.32
	*p*-value	0.0849^1^	0.3729^1^
		0.5788^2^	
Lean body mass (g), trunk	V2	22,137.88 ± 3,830.47	21,811.56 ± 3,549.12
	V5	21,803.45 ± 3,749.89	21,749.49 ± 3,594.10
	V2–V5	−334.43 ± 620.79	−62.07 ± 580.41
	*p*-value	0.0015^1^	0.4870^1^
		0.0619^2^	
Lean body mass (g), android	V2	3,172.15 ± 571.30	3,093.65 ± 572.82
	V5	3,121.43 ± 578.74	3,096.84 ± 580.68
	V2–V5	−50.73 ± 155.95	3.19 ± 143.93
	*p*-value	0.0464^1^	0.5805^2^
		0.2020^2^	
Lean body mass (g), gynoid	V2	7,184.05 ± 1,562.21	7,093.28 ± 1,466.82
	V5	7,064.95 ± 1,555.97	7,014.00 ± 1,407.88
	V2–V5	−119.10 ± 259.78	−79.28 ± 213.01
	*p*-value	0.0061^1^	0.0190^1^
		0.5027^2^	

1 Compared within groups using the Unpaired *t*-test or Wilcoxon signed-rank test.

2 Compared between groups using the Unpaired *t*-test or Wilcoxon rank-sum test.

In the arm area, lean body mass decreased significantly in both the intervention and control groups after the trial (*p* < 0.05), with no significant between-group difference. A similar finding was observed in the leg area. In the trunk and android areas, lean body mass decreased in both the intervention and control groups; however, statistical significance was only observed in the intervention group. Between-group comparisons revealed no significant difference. In the gynoid area, lean body mass decreased significantly in both the intervention and control groups after the trial (*p* < 0.05); however, there was no significant between-group difference.

### 3.4 Safety evaluation results

Safety evaluation was conducted using the Safety Analysis Set, and adverse reactions, vital signs, and laboratory test results were assessed for the initial 100 participants assigned to the trial (*n* = 50) and control (*n* = 50) groups. Approximately half of the participants in the trial (*n* = 26, 52.0%) and control (*n* = 26, 48%) groups had 40 adverse reactions each, with no statistically significant difference. One participant in the intervention group experienced a serious adverse reaction due to a traffic accident, requiring hospitalization; however, this incident was not directly related to the intervention food. In the intervention group, nine adverse reactions to viral infection (COVID-19), five cases of pharyngitis, and other symptoms such as headache and muscle pain were reported. In the control group, 11 adverse reactions to viral infection (COVID-19), eight cases of headache, and other symptoms, such as pharyngitis and diarrhea were reported. These incidents were related to normal food. Mild reactions were also reported, including five cases of pharyngitis and other symptoms, such as headache and muscle pain, which were unrelated to the intervention food. Regarding vital signs, no significant differences were observed in systolic blood pressure, diastolic blood pressure, pulse, or body temperature between trial periods or groups. Pre-and post-trial comparison of the hematological test results revealed a statistically significant decrease in hemoglobin level within the normal range in the intervention group (*p* < 0.05), and a slight decrease in the control group, with no statistical significance (Supplementary Table S1). Between-group comparisons revealed no significant difference. No statistically significant differences were observed in all the remaining items between trial periods or groups. Pre-and post-trial comparisons of the blood chemistry test results revealed a statistically significant increase in total protein and creatinine levels in the control group (*p* < 0.05), with no significant clinical difference. Between-group comparisons revealed no significant difference. Pre-and post-trial comparisons of the glucose levels revealed a decrease of 2.78 ± 10.67 mg/dL in the intervention group and a increase of 2.98 ± 10.07 mg/dL in the control group, with no statistically significant difference. Between-group comparisons revealed a more significant decrease in the intervention group than in the control group (*p* < 0.05). No statistically or clinically significant changes were observed for the other items (Supplementary Tables S1, S2).

## 4 Discussion

This RCT was conducted to confirm the effect of KP extract (SIRTMAX®) on body fat reduction among 100 participants randomly assigned equally to intervention and control groups in this double-blind study. The primary functional evaluation variables were changes in body fat mass and body fat percentage measured by DEXA. Body fat mass significantly decreased in the intervention group, compared with the control group. Body fat percentage did not change in the intervention group but increased in the control group, with no statistically significant difference between the intake groups. Lean body mass significantly decreased in both groups, with no significant between-group difference. In the intervention group, the decrease in lean body mass alongside a significant reduction in body fat mass may reflect an overall caloric deficit or mild catabolic state, despite efforts to maintain regular dietary and physical activity patterns. Interestingly, in the control group, lean body mass also decreased, despite a slight increase in body fat mass. This paradoxical finding may be attributed to body recomposition dynamics that are not solely dependent on fat loss. Specifically, age-related sarcopenia, physical inactivity, or subtle nutritional imbalances could have led to muscle mass loss even in the absence of caloric restriction. Furthermore, since the control group did not receive any active ingredient, their baseline metabolic trends may have continued unmitigated, reflecting ongoing age or lifestyle-related muscle atrophy. These patterns emphasize the complexity of body composition changes and support the need for multifaceted interventions targeting both fat reduction and muscle preservation in future studies. Regarding changes in body fat mass, body fat percentage, and lean body mass by region, no significant between-group differences in the arms and legs were observed; however, in the trunk, android, and gynoid areas, a more significant decrease in body fat mass was observed in the intervention group, with no significant between-group differences in body fat percentage and lean body mass. DEXA is an accurate method for measuring body fat percentage. It uses two low-energy X-rays to evaluate body composition, including body fat, lean body mass, and bone density, and shows relatively high accuracy and reproducibility when compared with other methods. In addition, it has the advantage of being able to explain the pattern of fat distribution by providing the results of scans for each part and the ratio of androids to gynoid (32–35). The significant decrease in body fat mass in the intervention group was determined through precise measurement using DEXA and through detailed measurement by area. The decrease in total body fat mass was believed to be because of a significant decrease in body fat mass in the intervention group in the trunk, android, and gynoid areas. However, lean body mass also decreased and the change in body fat percentage was not significant in the intervention group. Regardless, the effect in the intervention group was not inferior to that in the control group. Ideal health management is achieved when lean body mass is maintained or increased while body fat is decreased. During the study, participants were encouraged to maintain their existing diet and activity or exercise patterns; therefore, it is thought that lean body mass may have decreased somewhat in the process of decreasing body fat mass, and it may not have been easy to maintain or increase lean body mass. The phenomenon of simultaneous decrease in body fat mass and lean body mass can be interpreted in the context of various dietary methods or exercise programs. It is commonly suggested that the combination of resistance training, a calorie restriction diet, and high-protein diet can preserve or slightly increase lean body mass while reducing body fat (36, 37). Therefore, to effectively reduce body fat, while preserving muscle mass among lean body mass when losing weight, appropriate exercise and nutritional intake should be accompanied by appropriate body fat-reducing treatments.

Total fat area, subcutaneous fat area, visceral fat area, and VSR measured using abdominal CT were all significantly lower in the intervention group than in the control group. This shows that the intervention food had a significant effect on reducing abdominal fat, especially visceral fat, and this result is believed to be related to the significant decrease in body fat mass in the trunk, android, and gynoid regions. Body weight and BMI showed significant decreases in the intervention group after intake, but there was no significant change in the control group. Between-group comparison showed a more significant decrease in the intervention group, confirming the effect of the intervention food. Body weight was documented to have decreased continuously at each visit (4, 8, and 12 weeks) in the intervention group, and at the end of the 12th week, there was a more significant decrease in the intervention group than in the control group. BMI was confirmed to have a more significant decrease in the intervention group than in the control group at 8 and 12 weeks. This implies that the intervention food was effective in continuous weight loss and BMI reduction. Waist circumference continuously decreased at each visit (4, 8, and 12 weeks) after intake in both groups, and on the 12th week, a statistically significant decrease was observed before and after intake. Hip circumference decreased continuously at each visit (4, 8, and 12 weeks) after intake in both groups, and at the 12-week point, a statistically significant decrease was observed before and after intake, but no statistically significant difference was observed between the groups. Regarding waist to hip circumference ratio, no statistically significant difference was observed between trial periods or groups. The significant decrease in waist circumference indicated that the intervention food had a positive effect on abdominal and visceral fat reduction. This is in line with a previous study on body fat reduction (31) that was designed with a similar structure, which presented the effect of body fat mass reduction by reducing body fat in the trunk and android regions. Therefore, KP extract (SIRTMAX®) is believed to reduce body fat mass and weight through the same mechanism. Changes in the levels of total cholesterol, low-density lipoprotein (LDL) cholesterol, triglycerides, and high-density lipoprotein (HDL) cholesterol, which are related to blood lipids, were observed. In the intervention group, all parameters decreased after intake, and in the control group, only LDL cholesterol and HDL cholesterol decreased after intake; however, this was not statistically significant. No statistically significant differences were observed in leptin and adiponectin levels between the intervention and control groups; however, the decrease was greater in the intervention group. Leptin is mainly produced in white adipose tissue and plays a role in suppressing appetite and regulating energy consumption (38). Adiponectin is also secreted from adipose tissue; it improves insulin sensitivity, has an anti-inflammatory effect, is negatively related to body fat ratio, and is associated with visceral fat level; therefore, leptin levels tend to increase after weight loss (39). Although the level in the intervention group was not statistically significant, it did not show an inferior effect compared with that in the control group. In addition, the safety analysis showed no adverse reactions or clinically meaningful changes in the laboratory test values after the intervention food was consumed. Therefore, KP extract (SIRTMAX®) is considered safe for body fat reduction, even for long-term consumption. KP also known as ‘black ginger.’ has been reported to suppress weight gain, body fat accumulation, and glucose intolerance in obese mice (25–27), and it has been reported to increase energy consumption and fat utilization in previous RCTs (28, 29). It has also been confirmed to reduce total fat area and visceral fat area through RCTs conducted among adults that were overweight and obese (obesity stage 1) in Japan (28, 29). In the present study, statistically significant reductions in total fat area and visceral fat area were observed, similar to the results of the study conducted in Japan (28, 29). The significant decrease in body fat mass due to the intake of KP extract (SIRTMAX®), which was additionally confirmed in this study, is thought to be due to the significant decrease in body fat mass in the trunk, android, and gynoid regions, all of which are related to the abdominal area of the torso, and the decrease in body fat is believed to be caused by the decrease in visceral fat area owing to the consumption of KP extract (SIRTMAX®). Although KP extract (SIRTMAX®) significantly reduced body fat mass and body weight, no concurrent improvements were observed in lipid parameters such as cholesterol, triglycerides, LDL, or HDL levels. Therefore, while the reduction in visceral fat may suggest potential metabolic benefits, further studies are needed to determine whether KP intake leads to clinically meaningful improvements in cardiovascular or metabolic health markers.

In this study, several subgroup-related findings revealed nuanced effects that warrant further discussion. While overall body fat mass was significantly reduced in the intervention group compared to the control group, subgroup analyses by anatomical region (e.g., android, gynoid, trunk) showed varying levels of statistical significance. Specifically, although the trunk and android areas showed significant reductions in body fat mass, corresponding changes in body fat percentage and lean mass were not always consistent across these regions. Furthermore, in peripheral regions such as the arms and legs, the fat mass and percentage changes were minimal or non-significant. These findings suggest that the effect of KP extract (SIRTMAX®) may be more pronounced in central (visceral) fat regions rather than in appendicular fat compartments. Additionally, although the GLM analysis adjusted for sex, age, and family history of obesity (which were not evenly distributed between groups), some inconsistencies remained in the outcomes of leptin and adiponectin levels and body composition indices. This may be due to inherent biological variability or limitations in detecting the subtle effects in smaller subpopulations. These discrepancies highlight the importance of stratified analysis in future trials, especially regarding age, sex, metabolic status, and regional fat distribution patterns, which may modulate the response to KP extract (SIRTMAX®). It also emphasizes the need for longer-term studies with more rigorous control of lifestyle factors and dietary intake to discern more definitive trends across subgroups. In the intervention group, reductions were observed in both body fat mass (notably in the android and gynoid regions) and lean body mass (particularly in the arms, trunk, android, and gynoid regions). Notably, the degree of reduction in lean body mass appeared to be greater than that in fat mass in some regions. This finding raises an important concern regarding the desirability of KP extract (SIRTMAX®) as a standalone anti-obesity supplement, since preservation of lean body mass—especially skeletal muscle—is essential for metabolic health, physical function, and long-term weight maintenance. The greater decrease in lean body mass may have resulted from an overall caloric imbalance or insufficient protein intake, as dietary intake and physical activity were not strictly controlled. Therefore, while KP extract (SIRTMAX®) demonstrates promise in reducing visceral fat, it may not be appropriate to recommend its use in isolation for fat reduction without concurrent strategies aimed at preserving lean mass, such as resistance training and adequate protein consumption. Future studies should explore combined interventions and assess body composition changes in greater detail to determine the net clinical benefit of KP supplementation.

This study has several notable strengths. First, it was designed as a randomized, double-blind, placebo-controlled clinical trial, which is considered the gold standard in clinical research for minimizing bias and establishing causal inference. The use of block randomization and blinding across participants, investigators, and data analysts reduced selection and performance biases, enhancing the internal validity of the findings. Second, objective and precise measurement tools were employed, such as DEXA for total and regional body composition and abdominal CT for visceral and subcutaneous fat evaluation. These tools offer high sensitivity and specificity, particularly for detecting changes in fat distribution, which is crucial for evaluating the metabolic risks associated with obesity. Third, the study used a comprehensive set of primary and secondary endpoints, including biochemical markers (lipids, leptin, adiponectin), anthropometric data (BMI, waist/hip ratio), and regional fat assessments. This allowed for a holistic evaluation of the anti-obesity effects of KP extract (SIRTMAX®) beyond simple weight reduction. Fourth, the safety profile of KP extract (SIRTMAX®) was rigorously assessed through adverse event monitoring, laboratory testing, and vital sign tracking, and no significant safety issues were observed. This supports the feasibility of KP as a candidate for long-term use in obesity management.

Despite its strengths, this study has some limitations. First, imbalances in baseline characteristics, particularly age and family history of obesity, were observed between the intervention and control groups. Although these were statistically adjusted using covariate analysis, such imbalances may still introduce residual confounding factors, limiting the generalizability of the subgroup results. Second, while participants were instructed to maintain their habitual diet and physical activity, dietary intake and exercise were not strictly monitored or controlled. This lack of control introduces potential variability that could dilute or mask the true effect of the intervention, particularly on lean body mass and metabolic biomarkers. Third, although regional analyses of body fat provided valuable insight into fat distribution, the sample size may have been insufficient for detecting small but clinically relevant differences in subgroups, especially for regional lean mass and hormonal markers (e.g., leptin and adiponectin). Fourth, the study duration was limited to 12 weeks, which, although sufficient to detect short-term changes in body fat, is not adequate for evaluating long-term efficacy or sustainability of fat loss, preservation of lean mass, or metabolic outcomes such as insulin sensitivity and cardiovascular events. Lastly, this study was conducted in a Korean population with mild-to-moderate obesity (BMI 25–30 kg/m^2^), which may limit the applicability of the results to individuals with severe obesity, different ethnic backgrounds, or comorbidities that influence weight regulation.

In this study, we confirmed the effectiveness of KP extract (SIRTMAX®) in reducing body fat. If body fat can be effectively reduced, it can greatly improve metabolic function, increase insulin sensitivity of tissues, reduce the risk of diabetes and heart disease by lowering neutral fat levels, and it is expected to have various effects on promoting health (40–43). A variety of studies have been conducted on various supplements and substances. Various studies have demonstrated that probiotic supplementation significantly reduces BMI, body weight, and waist circumference in adults (30, 44). In other studies, cinnamon supplementation has been associated with significant reductions in body weight and BMI when compared to control groups, but no statistical significance has been observed in waist circumference (45). Furthermore, greater effects were observed at doses ≥3 g/day and in patients with polycystic ovary syndrome (PCOS). Another study revealed that Vitamin D supplementation significantly reduces BMI and waist circumference, but not body weight or fat mass, and was associated with doses of 5,000 IU/day or more for 16 weeks or less. Therefore, it is crucial to highlight the importance of considering dose, duration, and population-specific responses in future studies (45, 46).

The results of this study showed that body fat and visceral fat were significantly reduced; however, the standard for maintaining health is ensuring that muscle mass remains the same or increases during the process of body fat reduction. However, in the present study, muscle mass tended to decrease. In studies related to body fat reduction, the tendency for muscle loss has been shown to depend on various factors such as diet, exercise, and nutrition. When implementing a calorie-restricted diet to reduce body fat, the group that consumed high protein (1.6–2.4 g/kg) in a low-calorie state showed less muscle loss and more effective fat loss. However, there was no additional benefit when protein intake exceeded a certain level (47). Many studies that emphasized the method of combining a high-protein diet and an exercise program have addressed the importance of protein intake for preserving and increasing muscle mass during body fat reduction, and it has been reported that a high-protein diet plays an important role in maintaining muscle mass while increasing the rate of body fat reduction, and in particular, high protein intake together with an exercise program such as resistance exercise is the most effective combination for preventing muscle loss while reducing body fat (48, 49). During this RCT, the participants were asked to follow their usual diet and exercise routines; therefore, their protein intake may have been similar or even insufficient. Therefore, when taking the KP extract (SIRTMAX®) for the purpose of reducing body fat in the future, a high-protein diet and exercise should be combined to effectively reduce body fat and maintain muscle mass. If body fat is effectively reduced by KP extract (SIRTMAX®), adipose tissue plays an important role in metabolic regulation, which can improve insulin sensitivity and contribute to the prevention of diabetes. It can also reduce inflammation, help prevent diseases such as fatty liver, lower blood pressure, and improve cholesterol levels to reduce the risk of heart disease and stroke, thus providing various health benefits (50–52). Studies have also shown that reducing body fat can help improve body image and self-esteem, reduce depression and anxiety (53), improve athletic performance and overall quality of life, preserve muscle mass, and maintain physical function as we age (51).

## 5 Conclusion

This randomized, double-blind, placebo-controlled clinical trial demonstrated that KP extract (SIRTMAX®), taken daily for 12 weeks, significantly reduced total body fat mass and visceral fat in overweight and mildly obese Korean adults. The reductions were most pronounced in the trunk, android, and gynoid regions, indicating a targeted effect on central adiposity. Although lean body mass decreased slightly, no serious safety concerns were observed, and overall tolerability was high. These findings suggest that KP extract (SIRTMAX®) may serve as a safe and effective functional ingredient for body fat reduction and obesity management. Future long-term studies are needed to confirm sustained efficacy, assess preservation of muscle mass, and explore metabolic benefits across diverse populations.

## Data Availability

The original contributions presented in the study are included in the article/Supplementary material, further inquiries can be directed to the corresponding author.

## Supplementary material

The Supplementary material for this article can befound online at: https://www.frontiersin.org/articles/10.3389/fnut.2025.1576024/full#supplementary-material
