# Supplementary material for: Kaempferia parviflora Extract (SIRTMAX®) reduces body fat in adults who are overweight: a 12-week randomized, double-blind, placebo-controlled trial
Source: Front Nutr. 2025 Jun 25;12:1576024. doi: 10.3389/fnut.2025.1576024 (PMC12258047; doi:10.3389/fnut.2025.1576024)
Supplement: Supplementary file 1 [file Supplementary_file_1.docx]

**Table S1. Safety Analysis: Hematological tests and Blood chemistry test**

|  | | **Intervention group** | **Control group** | | | |
| --- | --- | --- | --- | --- | --- | --- |
| WBC (10^3^/µl), Mean±SD(n) | V1 | 6.27±1.44(50) | 6.18±1.47(50) | | | |
|  | V5 | 6.07±1.55(40) | 6.18±1.43(43) | | | |
|  | V5-V1 | -0.26±1.33(40) | -0.10±1.06(43) | | | |
|  | *p*-value | 0.2316^1^ | 0.5505^1^ | | | |
|  | *p*-value | 0.5516^2^ | | | | |
| RBC (10^3^/µl), Mean±SD(n) | V1 | 4.78±0.44(50) | 4.65±0.43(50) | | | |
|  | V5 | 4.75±0.42(40) | 4.63±0.39(43) | | | |
|  | V5-V1 | -0.02±0.21(40) | 0.01±0.17(43) | | | |
|  | *p*-value | 0.2915^1^ | 0.5580^1^ | | | |
|  | *p*-value | 0.2739^2^ | | | | |
| Hemoglobin (g/dl), Mean±SD(n) | V1 | 14.24±1.91(50) | 13.98±1.62(50) | | | |
|  | V5 | 13.95±1.97(40) | 13.70±1.41(43) | | | |
|  | V5-V1 | -0.17±0.61(40) | -0.12±0.56(43) | | | |
|  | *p*-value | 0.0199^1^ | 0.1672^1^ | | | |
|  | *p*-value | 0.5808^2^ | | | | |
| Hematocrit (%), Mean±SD(n) | V1 | 44.14±4.60(50) | 43.20±4.12(50) | | | |
|  | V5 | 43.94±4.84(40) | 43.18±3.73(43) | | | |
|  | V5-V1 | 0.03±1.73(40) | 0.29±1.77(43) | | | |
|  | *p*-value | 0.9204^1^ | 0.2962^1^ | | | |
|  | *p*-value | 0.5036^2^ | | | | |
| Platelets (10^3^/µl), Mean±SD(n) | V1 | 291.22±70.85(50) | 288.98±64.19(50) | | | |
|  | V5 | 288.18±72.20(40) | 283.28±65.49(43) | | | |
|  | V5-V1 | -4.45±28.23(40) | -4.47±25.64(43) | | | |
|  | *p*-value | 0.3249^1^ | 0.5220^1^ | | | |
|  | *p*-value | 0.7950^2^ | | | | |
| Glucose (mg/dl), Mean±SD(n) | V1 | 96.46±14.46(50) | 91.44±10.53(50) | | | |
|  | V5 | 90.80±12.27(40) | 94.21±11.34(43) | | | |
|  | V5-V1 | -2.78±10.67(40) | 2.98±10.07(43) | | | |
|  | *p*-value | 0.1081^1^ | 0.0697^1^ | | | |
|  | *p*-value | 0.0181^2^ | | | | |
| BUN (mg/dl), Mean±SD(n) | V1 | 12.86±3.96(50) | 13.24±3.81(50) | | | |
|  | V5 | 12.58±3.38(40) | 12.84±3.60(43) | | | |
|  | V5-V1 | -0.33±3.57(40) | -0.35±2.48(43) | | | |
|  | *p*-value | 0.5680^1^ | 0.3611^1^ | | | |
|  | *p*-value | 0.9721^2^ | | | | |
| Creatinine (mg/dl), Mean±SD(n) | V1 | 0.78±0.16(50) | 0.75±0.17(50) | | | |
|  | V5 | 0.79±0.17(40) | 0.75±0.17(43) | | | |
|  | V5-V1 | 0.02±0.09(40) | 0.03±0.08(43) | | | |
|  | *p*-value | 0.1059^1^ | 0.0345^1^ | | | |
|  | *p*-value | 0.9051^2^ | | | | |
| Total protein (g/dl), Mean±SD(n) | V1 | 7.08±0.33(50) | 7.02±0.31(50) | | | |
|  | V5 | 7.13±0.33(40) | 7.13±0.29(43) | | | |
|  | V5-V1 | 0.08±0.29(40) | 0.10±0.27(43) | | | |
|  | *p*-value | 0.0669^1^ | 0.0148^1^ | | | |
|  | *p*-value | 0.7479^2^ | | | | |
| Albumin (g/dl), Mean±SD(n) | V1 | 4.59±0.23(50) | 4.57±0.23(50) | | | |
|  | V5 | 4.59±0.22(40) | 4.57±0.23(43) | | | |
|  | V5-V1 | 0.03±0.19(40) | 0.04±0.20(43) | | | |
|  | *p*-value | 0.3573^1^ | 0.2182^1^ | | | |
|  | *p*-value | 0.8177^2^ | | | | |
| Total bilirubin (mg/dl), Mean±SD(n) | V1 | 0.42±0.24(50) | 0.41±0.22(50) | | | |
|  | V5 | 0.45±0.20(40) | 0.43±0.24(43) | | | |
|  | V5-V1 | 0.06±0.18(40) | 0.02±0.22(43) | | | |
|  | *p*-value | 0.0598^1^ | 0.5315^1^ | | | |
|  | *p*-value | 0.4591^2^ | | | | |
| AST (U/L),  Mean±SD(n) | V1 | 24.92±13.70(50) | 21.16±5.98(50) | | | |
|  | V5 | 23.38±9.19(40) | 22.67±8.73(43) | | | |
|  | V5-V1 | -1.88±13.38(40) | 1.26±7.95(43) | | | |
|  | *p*-value | 0.6528^1^ | 0.9509^1^ | | | |
|  | *p*-value | 0.7351^2^ | | | | |
| ALT (U/L),  Mean±SD(n) | V1 | 25.70±14.89(50) | 21.04±10.05(50) | | | |
|  | V5 | 22.08±12.65(40) | 20.79±12.22(43) | | | |
|  | V5-V1 | -2.75±9.90(40) | -0.35±10.88(43) | | | |
|  | *p*-value | 0.0867^1^ | 0.2730^1^ | | | |
|  | *p*-value | 0.8195^2^ | | | | |
| γ-GT (U/L),  Mean±SD(n) | V1 | 28.44±21.33(50) | 22.34±13.05(50) | | | |
|  | V5 | 27.75±17.47(40) | 24.63±19.41(43) | | | |
|  | V5-V1 | -2.68±9.68(40) | 1.91±13.06(43) | | | |
|  | *p*-value | 0.2843^1^ | 0.5925^1^ | | | |
|  | *p*-value | 0.7980^2^ | | | | |
| Total cholesterol (mg/dl), Mean±SD(n) | V1 | 215.12±43.80(50) | 206.18±34.84(50) | | | |
|  | V5 | 210.55±40.39(40) | 206.28±40.99(43) | | | |
|  | V5-V1 | -6.23±24.61(40) | 1.49±33.59(43) | | | |
|  | *p*-value | 0.0755^1^ | 0.6625^1^ | | | |
|  | *p*-value | 0.4041^2^ | | | | |
| Triglyceride (mg/dl), Mean±SD(n) | V1 | 169.72±198.40(50) | | | 143.50±93.39(50) | |
|  | V5 | 150.45±101.63(40) | | | 167.63±100.24(43) | |
|  | V5-V1 | -15.58±186.64(40) | | | 23.84±95.41(43) | |
|  | *p*-value | 0.8225^1^ | | | 0.0916^1^ | |
|  | *p*-value | 0.2904^2^ | | | | |
| HDL cholesterol (mg/dl), Mean±SD(n) | V1 | 51.84±13.62(50) | | 52.76±11.77(50) | | |
|  | V5 | 52.43±12.59(40) | | 52.00±11.99(43) | | |
|  | V5-V1 | -0.30±6.82(40) | | | | -1.40±7.53(43) |
|  | *p*-value | 0.7825^1^ | | | | 0.2310^1^ |
|  | *p*-value | 0.4905^2^ | | | | |
| LDL cholesterol (mg/dl), Mean±SD(n) | V1 | 140.04±38.70(50) | | | | 133.76±31.24(50) |
|  | V5 | 137.08±38.18(40) | | | | 131.51±36.44(43) |
|  | V5-V1 | -5.45±24.41(40) | | | | -0.72±29.98(43) |
|  | *p*-value | 0.1611^1^ | | | | 0.3709^1^ |
|  | *p*-value | 0.6550^2^ | | | | |
| ¹ Compared within groups using the Paired t-test or Wilcoxon signed-rank test ² Compared between groups using the Unpaired t-test or Wilcoxon rank-sum test | | | | | | |

**Table S2. Safety Analysis: Urine test results**

|  |  | Week 12/baseline (Week 1) | Normal, n (%) | Abnormal, n (%) |  |
| --- | --- | --- | --- | --- | --- |
| **Specific gravity** | Intervention group  (N=40) | Normal, n (%) | 30(75.00) | 3(7.50) |  |
|  |  | Abnormal, n (%) | 5(12.50) | 2(5.00) |  |
|  |  | *p*-value^1^ | 0.4795^1^ | |  |
|  | Control group  (N=43) | Normal, n (%) | 35(81.40) | 4(9.30) |  |
|  |  | Abnormal, n (%) | 4(9.30) | 0(0.00) |  |
|  |  | *p*-value^1^ | 1.0000^1^ | |  |
| **pH** | Intervention group  (N=40) | Normal, n (%) | 40(100.0) | 0(0.00) |  |
|  |  | Abnormal, n (%) | 0(0.00) | 0(0.00) |  |
|  |  | *p*-value^1^ | ^-^ | |  |
|  | Control group  (N=43) | Normal, n (%) | 42(97.67) | 1(2.33) |  |
|  |  | Abnormal, n (%) | 0(0.00) | 0(0.00) |  |
|  |  | *p*-value^1^ | - | |  |
| **Protein** | Intervention group  (N=40) | Normal, n (%) | 28(70.00) | 4(10.00) |  |
|  |  | Abnormal, n (%) | 5(12.50) | 3(7.50) |  |
|  |  | *p*-value^1^ | 0.7389^1^ | |  |
|  | Control group  (N=43) | Normal, n (%) | 34(79.07) | 1(2.33) |  |
|  |  | Abnormal, n (%) | 7(16.28) | 1(2.33) |  |
|  |  | *p*-value^1^ | 0.0339^1^ | |  |
| **Glucose** | Intervention group  (N=40) | Normal, n (%) | 40(100.0) | 0(0.00) |  |
|  |  | Abnormal, n (%) | 0(0.00) | 0(0.00) |  |
|  |  | *p*-value^1^ | - | |  |
|  | Control group  (N=43) | Normal, n (%) | 43(100.0) | 0(0.00) |  |
|  |  | Abnormal, n (%) | 0(0.00) | 0(0.00) |  |
|  |  | *p*-value^1^ | - | |  |
| **Blood (RBC)** | Intervention group  (N=40) | Normal, n (%) | 31(77.50) | 4(10.00) |  |
|  |  | Abnormal, n (%) | 4(10.00) | 1(2.50) |  |
|  |  | *p*-value^1^ | 1.0000^1^ | |  |
|  | Control group  (N=43) | Normal, n (%) | 33(76.74) | 3(6.98) |  |
|  |  | Abnormal, n (%) | 4(9.30) | 3(6.98) |  |
|  |  | *p*-value^1^ | 0.7055^1^ | |  |
| ^1^ *p*-value for McNemar’s test | | | | |  |
